# Commentary: Treatment Outcome of Different Chemotherapy in Patients With Relapsed or Metastatic Malignant Urachal Tumor

**DOI:** 10.3389/fonc.2022.864708

**Published:** 2022-05-30

**Authors:** Tibor Szarvas, Henning Reis

**Affiliations:** ^1^ Department of Urology, University Medicine Essen, University of Duisburg-Essen, Essen, Germany; ^2^ Department of Urology, Semmelweis University, Budapest, Hungary; ^3^ Dr. Senckenberg Institute of Pathology, University Hospital Frankfurt, Goethe University Frankfurt, Frankfurt am Main, Germany

**Keywords:** urachal cancer, chemotherapy, cisplatin, outcome, survival, molecular pathology

## Introduction

Urachal cancer (UrC) is a rare cancer derived from remnant urachal structures. UrC is often diagnosed in advanced tumor stages when curative therapy is not feasible resulting in moderate overall survival (OS) rates of approximately 50% (1). Due to the rarity of the tumor, prospective randomized clinical trials can hardly be conducted and therapeutic decision-making regarding systemic chemotherapy is therefore necessarily based on experience from case reports or smaller case series. In addition, targeted therapy approaches represent a further potential treatment option based on a biological rationale (2).

## Condensed Results of the Study

In the article by *Chen and colleagues*, 24 patients with relapsed or metastatic UrC were treated with different chemotherapy regimens and the clinical efficacy was retrospectively evaluated (3). Patients were grouped according to received therapy into platinum-based (n=16), taxane-based (n=11), and fluorouracil-based (n=15) cohorts resulting in overlapping groups. Another patient received tislelizumab monotherapy. In seven cases, molecular data obtained by next-generation sequencing (NGS) was available and at least in one patient PD-L1 expression analysis was conducted. Survival analyses revealed longer progression-free survival (PFS, p=0.032) in patients who received platinum-based chemotherapy, however a similar association could not be observed for patients’ OS.

## Discussion

Considering that prospective clinical trials are lacking and published data on the systemic treatment of UrC is sparse, any additional data on the systemic treatment of UrC represents a significant contribution to the clinical management of this disease. Therefore, we read with great interest the paper by Chen et al. and we appreciate the work they invested to collect and interpret their data. The most important finding of the study, however, resulted in rather surprising results as authors found platinum-based therapy to be superior regarding PFS to those of taxane and 5-FU based therapy regimens. This observation needs to be handled with critical caution for the following reasons:

First, the survival of UrC patients with platinum-based regimens has been reportedly poor with a median survival of less than one year [Molina JR, Cancer] (4). Accordingly, in a further case series of 20 UrC patients treated with various chemotherapy regimens, 5-FU combination with platinum proved to be superior to platinum combinations without 5-FU (5). Similar observations were found in our meta-analysis with 74 chemotherapy-treated UrC patients. The radiographic response rate was significantly higher in the 5-FU (44%) and the 5-FU+cisplatin (43%) groups compared to the platinum-based group having a response rate of only 9% (p=0.043). The combination of 5-FU+cisplatin also had the lowest progression rate and thus, seems to be have the best outcomes (5). Unfortunately, these data have not been referenced and discussed in the study by *Cheng and colleagues.*

Second, in small case series with heterogeneity in patients’ baseline characteristics, pretreatment and further treatment lines, time-to-event end-points (PFS, OS) are necessarily less accurate in the evaluation of therapy efficacy and may lead to inaccurate conclusions. In this study, 7 patients received previous adjuvant chemotherapy and further 12 patients were treated with second-line chemotherapy and further targeted therapies, which also largely contribute to treatment heterogeneity and makes PFS and OS less reliable end-points. Instead, primary radiographic response to each therapy line might be better suited as the study end-point.

Third, the data interpretation makes it difficult to follow the different therapy lines and grouping of patients. For example, according to Figure 2 the sum of patients in each treatment group is 42 (16x platinum-based, 15x 5-FU-based and 11x Taxane-based), which is higher than the number of patients in the whole study (n =24) suggesting that the same patients were apparently assigned to more than one therapy group (Figure 2). If our interpretation is correct, this suggests that some of the patients in the platinum group also received 5-FU in the same line of therapy and *vice versa*. In our opinion, therapy related and sequencing data should be provided on a patient individual level with corresponding radiographic response data in order to provide more transparent evaluation of data and to allow the use of data in further meta-analyses. In addition, more information on the used NGS methods and results would improve our understanding of the molecular background of UrC, especially as the authors describe tumor mutational burden in some cases, i.e. panel analysis of at least 1 mega base genetic code or greater had been sequenced.

## Conclusion

Currently available data rather support 5-FU and platinum combination or 5-FU-based regimens to be more effective in progressed UrC, but in lack of prospective clinical trials the level of evidence of this suggestion necessarily remains low and therefore further data from cases series are needed. This notion is supported by current data including an evidence-based guide for clinical practice and a population-based analysis of SEER data on survival benefit of chemotherapy in metastatic UrC (6, 7). However, in the latter publication, no information of type of chemotherapy was available in the SEER data set (7).

Regarding the commented publication, we think that clinical and follow-up data should be reported on an individual patient level as far as possible in order to improve comparability and allowing data pooling in later meta-analyses.

## Author Contributions

Both authors drafted a review the manuscript and approved the final version.

## Conflict of Interest

The authors declare that the research was conducted in the absence of any commercial or financial relationships that could be construed as a potential conflict of interest.

## Publisher’s Note

All claims expressed in this article are solely those of the authors and do not necessarily represent those of their affiliated organizations, or those of the publisher, the editors and the reviewers. Any product that may be evaluated in this article, or claim that may be made by its manufacturer, is not guaranteed or endorsed by the publisher.
